# Insights on *Lulworthiales* Inhabiting the Mediterranean Sea and Description of Three Novel Species of the Genus *Paralulworthia*

**DOI:** 10.3390/jof7110940

**Published:** 2021-11-06

**Authors:** Anna Poli, Valeria Prigione, Elena Bovio, Iolanda Perugini, Giovanna Cristina Varese

**Affiliations:** Mycotheca Universitatis Taurinensis, Department of Life Sciences and Systems Biology, University of Torino, Viale Mattioli 25, 10125 Torino, Italy; anna.poli@unito.it (A.P.); elena.bovio@inrae.fr (E.B.); jolanda.perugini@unito.it (I.P.); cristina.varese@unito.it (G.C.V.)

**Keywords:** marine fungi, novel lineages, phylogeny, genetic markers

## Abstract

The order *Lulworthiales*, with its sole family *Lulworthiaceae*, consists of strictly marine genera found on a wide range of substrates such as seagrasses, seaweeds, and seafoam. Twenty-one unidentified *Lulworthiales* were isolated in previous surveys aimed at broadening our understanding of the biodiversity hosted in the Mediterranean Sea. Here, these organisms, mostly found in association with *Posidonia oceanica* and with submerged woods, were examined using thorough multi-locus phylogenetic analyses and morphological observations. Maximum-likelihood and Bayesian phylogeny based on nrITS, nrSSU, nrLSU, and four protein-coding genes led to the introduction of three novel species of the genus *Paralulworthia*: *P. candida*, *P. elbensis*, and *P. mediterranea*. Once again, the marine environment is a confirmed huge reservoir of novel fungal lineages with an under-investigated biotechnological potential waiting to be explored.

## 1. Introduction

The increasing interest in marine fungi continues to widen our knowledge of marine biodiversity. So far, more than 1900 species inhabiting the oceans have been described (www.marinefungi.org); however, most of the fungal diversity, estimated to exceed 10,000 taxa [1], is yet to be uncovered. Marine habitats and substrates, both biotic and abiotic, are continuously being explored worldwide, leading to the discovery of new marine fungal lineages.

Sordariomycetes are one of the classes mostly detected in the sea (www.marinefungi.org) and include several orders, namely, *Coronophorales*, *Chaetosphaeriales*, *Diaporthales*, *Hypocreales*, *Koralionastetales*, *Lulworthiales*, *Magnaporthales*, *Microascales*, *Ophiostomatales*, *Phyllachorales*, *Savoryellales*, *Sordariales*, *Tirisporellales*, *Torpedosporales*, and *Xylariales* [2,3]. *Lulworthiales* and *Koralionastetales*, recently placed in the new subclass *Lulworthiomycetidae* [4,5], consist of exclusively marine taxa. The order *Lulworthiales*, with its sole family *Lulworthiaceae*, was introduced on the basis of morphological characters and phylogenetic analyses built upon nrSSU and nrLSU partial sequences to accommodate the polyphyletic genera *Lulworthia* and *Lindra* [6,7]. The family *Lulworthiaceae* consists of strictly marine genera—including *Cumulospora*, *Halazoon, Hydea*, *Kohlmeyerella*, *Lulwoana*, *Lulwoidea, Lulworthia*, *Lindra*, *Matsusporium*, *Moleospora*, *Rostrupiella*, *Sammeyersia*, and the recently described genus *Paralulworthia*—that are distributed worldwide and found on a variety of substrates such as submerged wood, seaweeds, seagrasses, seafoam, and aquatic plants [4,8,9]. Members of this family are well-known cellulase producers and can break down complex lignocellulose compounds, thus contributing to the recycling of nutrients [10]. Morphologically, they are characterized by ascomata subglobose to cylindrical, 8-spored asci, cylindrical to fusiform and filamentous ascospores with end chambers filled with mucus (the latter character is missing in *Lindra*) [6,11].

Twenty-one unidentified *Lulworthiales* were isolated in previous surveys aimed at broadening our knowledge on the underwater fungal diversity of the Mediterranean Sea: sixteen isolates were obtained from the seagrass *Posidonia oceanica* [12], three from submerged wood [13], and two from seawater contaminated by oil spills. Traditionally, the identification of fungi at species level is based on the description of sexual and/or asexual reproductive structures. However, it is not unusual to deal with marine fungi that neither sporulate nor develop reproductive structures in axenic culture. Therefore, the identification of sterile mycelia must rely on molecular data [4,14,15,16,17]. In light of valuable biotechnological exploitations of marine fungi, correct taxonomic placement of sterile mycelia is necessary.

With this study, we tried to provide a better phylogenetic placement of the Mediterranean *Lulworthiales* by applying a combined multi-locus molecular phylogeny. Following phylogenetic inference and morphological insights, the three new species, *Paralulworthia candida*, *Paralulworthia elbensis*, and *Paralulworthia mediterranea*, were hereunder proposed.

## 2. Materials and Methods

### 2.1. Fungal Isolates

The isolates analysed in this study were recovered during previous surveys from the Mediterranean Sea in Italy. In detail, two isolates were derived from a site chronically contaminated by an oil spill in Gela (Caltanissetta, Italy) [17], three from submerged woods sampled in the Marine Protected Areas Island of Bergeggi (Savona, Italy) [13], and sixteen from twelve plants of *P. oceanica* collected in the coastal waters off the Elba Island (Livorno, Italy) from two sampling sites, Ghiaie and Margidore [12] (Table 1). The strains were isolated on Corn Meal Agar medium supplemented with sea salts (CMASS; 3.5% *w*/*v* sea salt mix, Sigma-Aldrich, Saint Louis, MO, USA, in ddH_2_O), and are currently preserved at the Mycotheca Universitatis Taurinensis (MUT), Italy.

### 2.2. Morphological Analysis

The strains were grown on Malt Extract Agar seawater (MEASW; 20 g malt extract, 20 g glucose, 2 g peptone, 20 g agar—Sigma-Aldrich, Saint Louis, MO, USA—in 1 L of seawater) for one month at 21 °C prior to inoculation in triplicate onto new MEASW Petri dishes (9 cm Ø). Plates were incubated at 15 and 21 °C. The colony growth was monitored periodically for 28 days, while macroscopic and microscopic features were assessed at the end of the incubation period.

Efforts to induce sporulation were carried out by applying sterile pieces of *Quercus ruber* cork and *Pinus pinaster* wood (species autochthonous to the Mediterranean area) on three-week-old fungal colonies [18]. Plates were further incubated for four weeks at 21 °C. Cork and wood specimens were transferred to 50 mL tubes containing 20 mL of sterile seawater. Samples were incubated at 21 °C for a minimum of three months up to nine months.

Morphological structures were observed, and images captured using an optical microscope (Leica DM4500B, Leica microsystems GmbH, Wetzlar, Germany) equipped with a camera (Leica DFC320, Leica microsystems GmbH, Wetzlar, Germany).

### 2.3. DNA Extraction, PCR Amplification, and Data Assembling

Fresh mycelium carefully scraped from MEASW plates was transferred to a 2 mL Epperndorf tube and disrupted by a MM400 tissue lyzer (Retsch GmbH, Haan, Germany). Genomic DNA was extracted following the manufacturer’s instructions of a NucleoSpin kit (Macherey Nagel GmbH, Duren, DE, USA). The quality and quantity of DNA were measured spectrophotometrically (Infinite 200 PRO NanoQuant; Tecan, Männedorf, Switzerland), and DNA samples were stored at −20 °C.

The partial sequences of seven genetic markers were amplified by PCR. Primer pairs ITS1/ITS4 [19], LR0R/LR7 [20], and NS1/NS4 [19] were used to amplify the internal transcribed spacers, including the 5.8S rDNA gene (nrITS), 28S large ribosomal subunit (nrLSU), and 18S small ribosomal subunit (nrSSU). The translation elongation factor (TEF-1α), the β-tubulin (β-TUB), and the largest and second-largest subunits of RNA polymerase II (RPB1 and RPB2) were amplified by using the following primer pairs: EF-dF/EF-2218R [21], Bt2a/Bt2b [22], RPB1Af/RPB1Cr [23], and fRPB2-5F/fPB2-7R [24]. Reaction mixtures consisted of 20–40 ng DNA template, 10× PCR Buffer (15 mM MgCl_2_, 500 mM KCl, 100 mM Tris-HCl, pH 8.3), 200 µM each dNTP, 1 μM each primer, and 2.5 U Taq DNA Polymerase (Qiagen, Chatsworth, CA, USA) in 50 μL final volume. Negative controls with no DNA template were included. For problematic cases, additional MgCl_2_, BSA, and/or 2.5% DMSO were supplied. Amplifications were run in a T100 Thermal Cycler (Bio-Rad, Hercules, CA, USA) programmed as described in Table 2.

Amplicons and a GelPilot 1 kb, plus a DNA Ladder, were visualized on a 1.5% agarose gel stained with 5 mL 100 mL^−1^ ethidium bromide. PCR products were purified and sequenced at the Macrogen Europe Laboratory (Madrid, Spain). The resulting Applied Biosystem (ABI) chromatograms were inspected, trimmed, and assembled to obtain consensus sequences using Sequencer 5.0 (GeneCodes Corporation, Ann Arbor, MI, USA; http://www.genecodes.com). Newly generated sequences were deposited in GenBank with the accession numbers reported in Table 1 and Table S1.

### 2.4. Sequence Alignment and Phylogenetic Analysis

**Figure 1:**
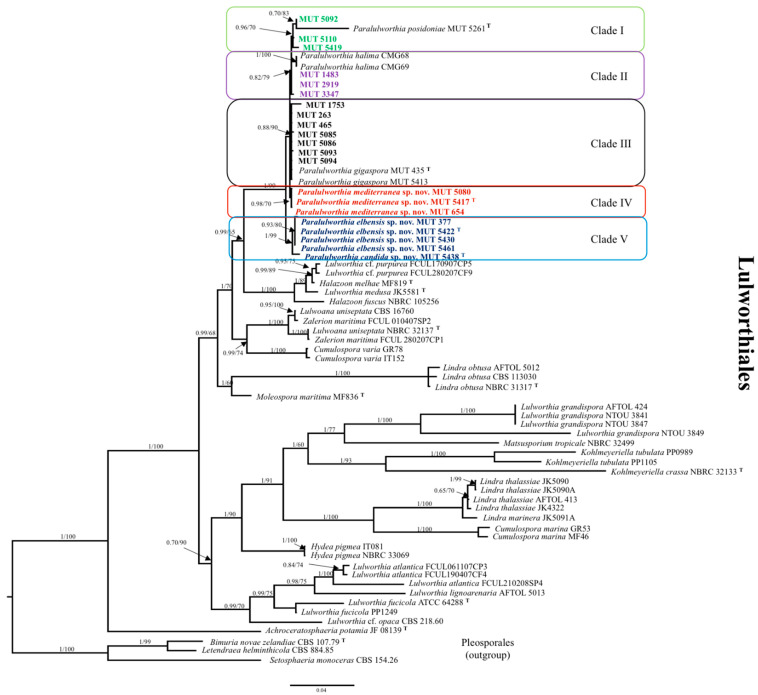
Phylogenetic inference based on a combined nrITS, nrSSU, and nrLSU dataset. The tree is rooted to three species of Pleosporales. Different colors indicate the belonging to different clades; in bold the strains analyzed in this study. Branch numbers indicate BYPP and BS values; **^T^** = Type Strain; Bar = expected changes per site (0.04).

A dataset consisting of nrSSU, nrITS, and nrLSU was assembled on the basis of BLASTn results and of the available phylogenetic studies focused on *Lulworthiales*, *Lulworthiaceae*, and *Lulworthia* [5,6,7,9,11,25,26]. Reference sequences were obtained from GenBank (Table 1). Sequences were aligned using MUSCLE (default conditions for gap openings and gap extension penalties), implemented in MEGA X (Molecular Evolutionary Genetics Analysis), visually inspected, and manually trimmed to delimit and discard ambiguously aligned regions. Alignments were concatenated into a single data matrix with SequenceMatrix [27] since no incongruence was observed among single-loci phylogenetic trees. The best evolutionary model under the Akaike Information Criterion (AIC) was determined with jModelTest 2 [28]. Phylogenetic inference was estimated using Maximum Likehood (ML) and Bayesian Inference (BI) criteria. The ML analysis was generated using RAxML v. 8.1.2 [29] under GTR + I + G evolutionary model and 1000 bootstrap replicates. Support values from bootstrapping runs (BS) were mapped on the global best tree using the “-f a” option of RAxML and “-x 12345” as a random seed to invoke the novel rapid bootstrapping algorithm. BI was performed with MrBayes 3.2.2 [30] with the same substitution model (GTR + I + G). The alignment was run for 10 million generations with two independent runs each, containing four Markov Chains Monte Carlo (MCMC) and sampling every 100 iterations. The first 25% of generated trees were discarded as “burn-in”. A consensus tree was generated using the “sumt” function of MrBayes and Bayesian posterior probabilities (BYPP) were calculated. Consensus trees were visualized in FigTree v. 1.4.2 (http://tree.bio.ed.ac.uk/software/figtree). Three species of Pleosporales, namely, *Bimuria novae-zelandiae*, *Letendraea helminthicola*, and *Setosphaeria monoceras*, were used as an outgroup, as indicated in previous studies [26]. Due to a topological similarity of the two resulting trees, only Bayesian analysis with BS and BYPP values was reported (Figure 1).

Following, a new phylogenetic analysis was conducted, focusing only on the under investigation, whose relationships were unclear. To this aim, TEF-1α, β-TUB, RPB1, and RPB2 sequences were added to the restricted dataset (Table S1). Alignments and multi-loci phylogeny were conducted as described above. *Lulworthia* cf. *purpurea*, *Halazoon melhae*, *Lulworthia medusa*, and *Cirrenalia fusca* were used as the outgroup.

Sequence alignments and phylogenetic trees were deposited in TreeBASE (http://www.treebase.org, submission number S28658 and S28660).

## 3. Results

### 3.1. Phylogenetic Inference

Preliminary analyses carried out individually with nrITS, nrSSU, and nrLSU revealed no incongruence in the topology of the single-loci trees. The combined three-markers dataset—built on the basis of the BLASTn results and available phylogenetic studies [5,6,7,9,11,25,26]—consisted of 69 taxa (including MUT strains) that represented 15 genera and 29 species (Table 1). A total of 148 sequences (21 nrITS, 21 nrSSU, 21 nrLSU, 20 TEF-1α, 24 β-TUB, 17 RPB1, and 24 RPB2) were newly generated, whereas 115 were obtained from GenBank.

The dataset combining nrSSU, nrITS, and nrLSU had an aligned length of 2166 characters, of which 1130 were conserved, 355 were parsimony-uninformative, and 681 parsimony-informative (TL = 2511, CI = 0.530999, RI = 0.776449, RC = 0.412294, HI = 0.469001). The strains investigated formed a monophyletic lineage (BYPP = 0.99; BS = 65%), with its closest relatives being *Lulworthia* cf. *purpurea*, *Halazoon mehlae*, *Lulworthia medusa*, and *H. fuscus* (Figure 1). Within this new group, five clades could be observed, as follows: MUT 5092, MUT 5110, and MUT 5419 clustered together with *Paralulworthia posidoniae*; MUT 1483 MUT 2919 and MUT 3347 were identified as *Paralulworthia halima* by performing BLASTn analysis of nrITS, nr SSU, and nrLSU relative to the three strains (nucleotide similarity between 99% and 100%); MUT 263, MUT 465, MUT 1753, MUT 5085, MUT 5086, MUT 5093, and MUT 5094, grouped together with *Paralulworthia gigaspora*; the fourth clade included MUT 654, MUT 5080, and MUT 5417 and appeared to support a new species of *Paralulworthia*; likewise, MUT 377, MUT 5422, MUT 5430, MUT 5438, and MUT 5461 formed the fifth clade.

The supplemental dataset, implemented with the addition of TEF-1α, RPB1, RPB2, and β-TUB sequence data relative to the strains investigated, had an aligned length of 4623 characters, of which 4322 were conserved, 144 were parsimony-uninformative, and 157 were parsimony-informative (TL = 351, CI = 0.800971, RI = 0.909292, RC = 0. 728316, HI = 0.199029). The segregation of the strains was more evident, confirming the conclusions previously drawn. In detail, by inspecting the tree, rooted to the group consisting of *L.* cf. *purpurea*, *H. mehlae*, *L. medusa*, and *H. fuscus*, it was possible to distinguish two groups in the genus *Paralulworthia sensu lato*: (a) the cluster (BYPP = 1.00; BS = 98%) that included the new species, *Paralulworthia mediterranea* (MUT 654, MUT 5080, and MUT 5417); and (b) the cluster consisting of MUT 377, MUT 5422, MUT 5430, MUT 5438, and MUT 5461 (BYPP = 0.83; BS = 70%) that represented two additional novel species of the same genus (Figure S1), namely, *Paralulworthia elbensis* (MUT 377, MUT 5422, MUT 5438) and *Paralulworthia candida* (MUT 5430).

### 3.2. Taxonomy

#### 3.2.1. *Paralulworthia mediterranea* sp. nov. A. Poli, E. Bovio, G.C. Varese and V. Prigione

**Figure 2:**
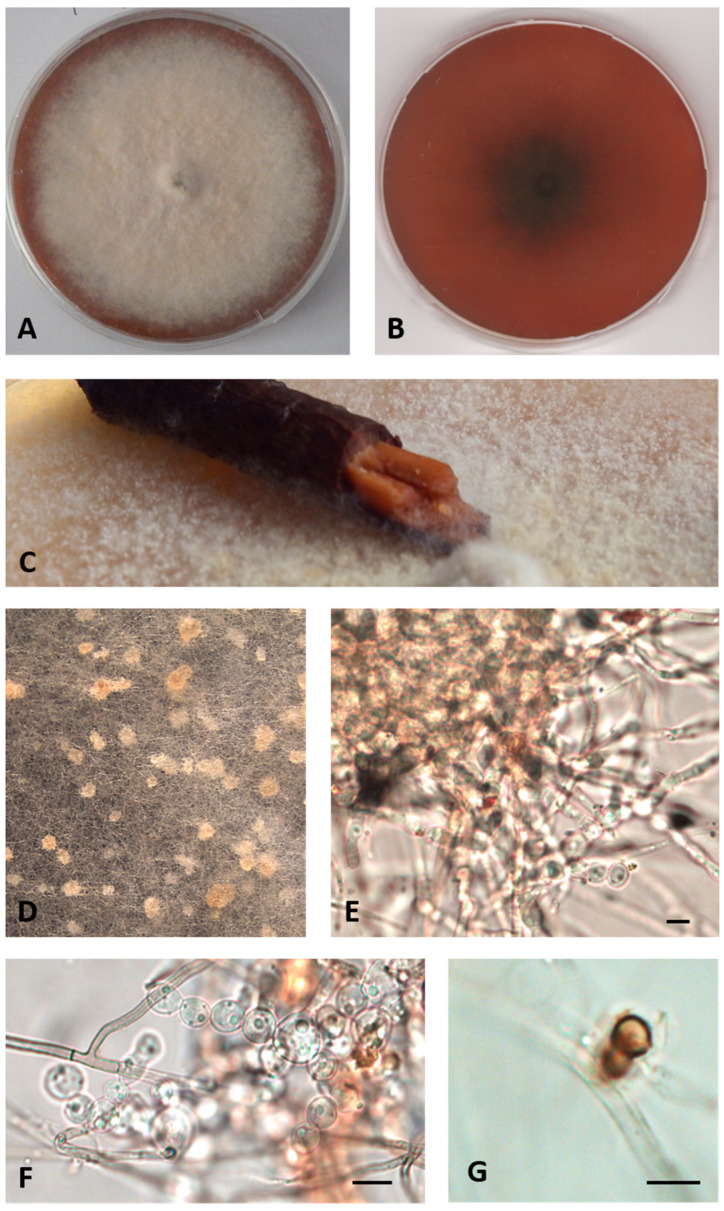
*Paralulworthia mediterranea* sp. nov. MUT 5417. 28-day-old colony at 21 °C on MEASW (**A**) and reverse (**B**); early colonization of *Pinus pinaster* wood (**C**); mycelium with bulbils (**D**); particular of a bulbil (**E**); swollen hyphae (**F**); two-celled chlamydospore (**G**). Scale bar: 10 μm.

- MYCOBANK: MB841118
- Type: Italy, Tuscany, the Mediterranean Sea, Elba Island (Livorno), Ghiaie, 3–5 m depth, 42°49′04″ N, 10°19′20″ E, from *Posidonia oceanica* roots, March 2010, R. Mussat-Sartor and N. Nurra, MUT 5417 holotype, living culture permanently preserved in metabolically inactive state by deep-freezing at MUT.
- Additional material examined: Italy, Tuscany, the Mediterranean Sea, Elba Island (Livorno), Ghiaie, 3–5 m depth, 42°49′04″ N, 10°19′20″ E from *Posidonia oceanica* rhizomes, March 2010, R. Mussat-Sartor and N. Nurra, MUT 654. Italy, Tuscany, the Mediterranean Sea, Elba Island (Livorno), Ghiaie, 3–5 m depth, 42°49′04″ N, 10°19′20″ E from *Posidonia oceanica* rhizomes, March 2010, R. Mussat-Sartor and N. Nurra, MUT 5080.
- Etymology: In reference to the Mediterranean Sea.
- Description: Growing actively on *Pinus pinaster* wood and *Quercus ruber* cork, more markedly on the first. *Hyphae* 2.4–4 μm wide, septate, from hyaline to dematiaceous. *Chlamydospores* light brown 4–5 × 5–6 μm, unicellular or two-celled often present. *Bulbils* on the colony surface single or in group, pale yellow or cream colored, becoming ochre with age, nearly spherical, 150–400 μm diameter, formed by swollen cells (10–15 μm diameter) (Figure 2).
- Sexual morph not observed. Asexual morph with differentiated conidiogenesis not observed.
- Colony description: Colony growing on MEASW, reaching 57–70 mm diameter after 14 days at 21 °C, mycelium feltrose, becoming granular with age due to the presence of bulbils, with irregular edges, beige, sometimes with greyish shades at the edges; reverse from amber to dark orange. A yellowish brown colored diffusible pigment was often present (Figure 2).

#### 3.2.2. *Paralulworthia candida* sp. nov. A. Poli, E. Bovio, V. Prigione and G.C. Varese

**Figure 3:**
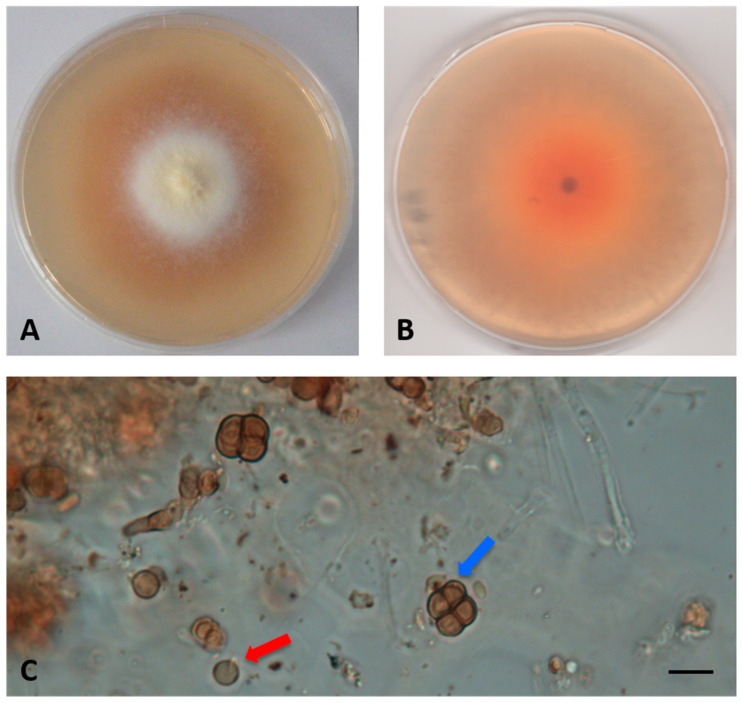
*Paralulorthia candida* sp. nov. MUT 5430. 28-day-old colony at 21 °C on MEASW (**A**) and reverse (**B**); unicellular (red arrow) and eight-cellular chlamydospores—blue arrow, (**C**). Scale bar: 10 μm.

- MYCOBANK: 841116
- Type: Italy, Tuscany, the Mediterranean Sea, Elba Island (Livorno), Ghiaie, 3–5 m depth, 42°49′04″ N, 10°19′20″ E, from *Posidonia oceanica* roots, March 2010, R. Mussat-Sartor and N. Nurra, MUT 5430 holotype, living culture permanently preserved in metabolically inactive state by deep-freezing at MUT.
- Etymology: In reference to the colony color.
- Description: Poor colonization of *Pinus pinaster* wood and *Quercus ruber* cork. *Hyphae* 2.2–4.2 μm wide, septate, hyaline. *Chlamydospores* abundant, brown, globose, or subglobose, from unicellular (5–7 × 5–8 μm) to eight-cellular (8–13 μm diameter), in the shape of a sarcina (Figure 3).
- Sexual morph not observed. Asexual morph with differentiated conidiogenesis not observed.
- Colony description. Growing on MEASW, reaching 27–32 mm diameter after 14 days at 21 °C, mycelium floccose, white with yellowish shades in the center, submerged edges giving a beige halo to the colony; reverse light orange. A pinkish colored diffusible pigment present (Figure 3).

#### 3.2.3. *Paralulworthia elbensis* sp. nov. A. Poli, E. Bovio, V. Prigione and G.C. Varese

**Figure 4:**
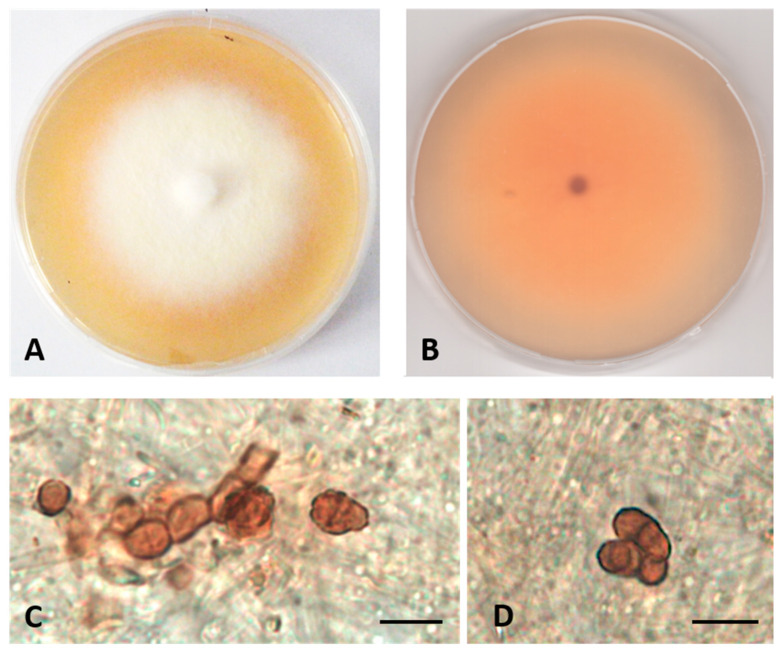
*Paraulworthia elbensis* sp. nov. MUT 5422. 28-day-old colony at 21 °C on MEASW (**A**) and reverse (**B**); chlamidospores in chain (**C**); multicellular chlamydospores (**D**). Scale bar: 10 μm.

- MYCOBANK: MB841117
- Type: Italy, Tuscany, the Mediterranean Sea, Elba Island (Livorno), Margidore,14–15 m depth, 42°45′29″ N, 10°18′24″ E, from *Posidonia oceanica* roots, March 2010, R. Mussat-Sartor and N. Nurra, MUT 5422 holotype, living culture permanently preserved in metabolically inactive state by deep-freezing at MUT.
- Additional material examined: Italy, Tuscany, the Mediterranean Sea, Elba Island (Livorno), Ghiaie, 3–5 m depth, 42°49′04″ N, 10°19′20″ E, from *Posidonia oceanica* roots, March 2010, R. Mussat-Sartor and N. Nurra, MUT 377. Italy, Tuscany, the Mediterranean Sea, Elba Island (Livorno), Margidore,14–15 m depth, 42°45′29″ N, 10°18′24″ E, from *Posidonia oceanica* roots, March 2010, R. Mussat-Sartor and N. Nurra, MUT 5438. Italy, Tuscany, the Mediterranean Sea, Elba Island (Livorno), Margidore, 14–15 m depth, 42°45′29″ N, 10°18′24″ E, from *Posidonia oceanica* roots, March 2010, R. Mussat-Sartor and N. Nurra, MUT 5461.
- Etymology: In reference to the location of isolation.
- Description: Poor colonization of *Pinus pinaster* wood and *Quercus ruber* cork. *Hyphae* 2.6–4.5 μm wide, septate, hyaline. *Chlamydospores* abundant, brown, single or in chains, from globose to ellipsoidal, unicellular (5–7 × 6–7 μm) or multicellular (8–11 × 9–12 μm diameter) (Figure 4).
- Sexual morph not observed. Asexual morph with differentiated conidiogenesis not observed.
- Colony description. Growing on MEASW, reaching 35–37 mm diameter after 14 days at 21 °C, mycelium feltrose, white with yellowish shades, submerged edges; reverse light orange (Figure 4).

## 4. Discussion

The morphological description of the strains object of this study was complicated by the absence of reproductive structures in pure cultures, thus making the description of diagnostic features amongst the newly recognized lineages impossible. For the same reason, the morphological comparison between the strains in analysis and the recently accepted species was unfeasible.

For a better characterization of these fungi, we used a culture medium supplemented with seawater to mimic their natural environment. Indeed, it is known that only the addition of seawater supports a measurable growth of vegetative mycelium [16,31]. Placing wood and cork specimens on the colonies’ surface, followed by their transfer into seawater to induce sporulation, was not successful. In fact, despite wood colonization, only chlamydospores were produced. Strictly vegetative growth is now a recognized feature of a relatively high percentage of marine fungi isolated from different substrates [31,32,33,34,35,36]. This may be due to the lack of appropriate environmental conditions these organisms are adapted to (e.g., high salinity, low temperature, high hydrostatic pressure, etc.) or simply to the fact that the dispersal of sterile marine fungi relies on hyphal fragments and/or resistance structures. In addition, it must be considered that, in fungi, sexual reproduction is controlled by the mating-type (MAT1) locus and that, contrary to homothallic self-fertile filamentous ascomycetes, mating in heterothallic self-sterile species is possible only between strains morphologically indistinguishable with a different idiomorph at the MAT1 locus [37,38,39]. As a consequence of fungal sterility, the identification of the 21 *Lulworthiales* was achieved with the help of molecular and phylogenetic data. Notwithstanding, the lack of sporulation in the strains identified as *P. gigaspora* and *P. posidoniae* puzzled us since sexual structures had previously been observed and described [8]. A possible reason for this behavior may be found in the strain-specific behavior of a homothallic species: homothallism, may, in fact, be a necessary but not sufficient condition for self-fertility to occur. Strains may be more or less sensitive to a range of conditions such as light, temperature, or salinity. Alternatively, an idiomorph may be eliminated via homologous recombination, as demonstrated in *Chromocrea spinulosa*—which exhibits both homothallic and heterothallic behaviour [37]—or, as seen in some species such as *Thielaviopsis cerberus* [40], it may display a unidirectional mating-type switching.

The inspection of the phylogenetic tree, based on the three ribosomal genes (nrITS, nrLSU, and nrSSU), highlights the presence of five hypothetical clades (Figure 1). In detail, three strains grouped with *P. posidoniae*, three clustered with *P. halima*, and seven seemed affiliated with *P. gigaspora*. The remaining formed two well-supported clusters that did not encompass any known fungus, indicating the presence of new lineages. To clarify and be certain of the relations among the species, a new dataset focusing on the five clades and their closest relatives (*L.* cf. *purpurea*, *H. mehlae*, *L. medusa*, and *H. fuscus*) was built with the addition of four protein-coding genes, namely, TEF-1α, RPB1, RPB2, and βTUB (Figure S1). Given the presence of intron regions, which can evolve at a faster rate compared to ribosomal regions, protein-coding genes are more informative and can be employed to improve phylogenetic accuracy, providing a clear species-level identification [23,41]. Indeed, the phylogenetic tree constructed upon seven markers points out the presence of two groups: (a) the cluster that includes the *P. gigaspora*, *P. halima*, *P. posidoniae*, and the newly found *P. mediterranea* clades; and (b) the cluster that consists of two additional new species, *P. candida* and *P. elbensis*. Besides the ultimate scope of our investigation, all the newly generated protein-coding sequences greatly enrich the public databases, thus increasing the availability of molecular data for researchers dealing with this group of fungi.

One could argue and contest the fact the introduction of novel species is based on molecular and phylogenetic data only. However, we followed the key recommendations outlined by Jeewon and Hyde [42]. As indicated by the authors, all the ITS sequences (including 5.8S) analyzed are longer than the minimum requirement of 450 base pairs; the tree is based on genes with strong phylogenetic signals and is statistically supported and includes the minimum number of closely related taxa of the same genus (Figure 1). Finally, reliable statistical support for each new clade (at least 60% BS or 0.9 BYPP) confirms taxa distinctiveness (Figure 1 and Figure S1).

The order Lulworthiales, with its sole family Lulworthiaceae, was erected to accommodate the genera *Lulworthia* and *Lindra*, once considered part of *Halosphaeriales* (fam. *Halosphaeriaceae*) [6,7], and was then moved to the new subclass *Lulworthiomycetidae* [5]. The polyphyletic nature of these two genera initially confused taxonomists, although nowadays, following a number of revisions [7,8,25,26], it is broadly accepted and is once more demonstrated in our investigation (Figure 1).

*Lulworthiaceae* are found in cold, temperate, and tropical waters in association with woods, seaweeds, seagrasses, and seafoam [4,8,9]. Likewise Goncalves et al. [9], the strains of *P. halima*, produced only chlamydospores and derived from submerged woods, indicating a preference of this species for such a substrate. Two strains of *P. gigaspora* were isolated from an oil spill, while the rest were associated with the seagrass *P. oceanica*. Members of *Lulworthiaceae* are known saprobes (http://www.funguild.org) and cellulases producers [10]. Considering the substrates of isolation and the production of lignocellulosic enzymes, we can hypothesize a lignicolous nature of the newly identified species. The retrieval of two strains of *P. gigaspora* from an oil spill reinforces the idea that these organisms can break down complex lignocellulose and recalcitrant compounds, thus contributing to the recycling of nutrients and possibly degrading contaminants such as polycyclic aromatic hydrocarbons (PAH). Interestingly, Paço and collaborators demonstrated the ability of a strain of *Zalerion maritimum* to degrade polyethylene [43], suggesting a key role of *Lulworthiaceae* in offering a solution to microplastic pollution. Further experiments will be necessary to assess the full degradative potential of these organisms that could be harnessed for bioremediation purposes.

## 5. Conclusions

In conclusion, the retrieval of fungi affiliated with *Lulworthiales*—together with the introduction of the novel species *Paralulworthia candida*, *Paralulworthia elbensis*, and *Paralulworthia mediterranea*—greatly contributes to improving our knowledge on this strictly marine order and to step-by-step unveiling the fungal communities hosted in the Mediterranean Sea.

Due to the extraordinary biotechnological potential demonstrated by marine fungi, a few strains described in this paper are currently being investigated for the production of novel bioactive molecules. However, we must bear in mind that the applicative value of these organisms depends on their identification at the species level, safe long-term preservation, and on the accessibility guaranteed by the public collections of biological resources.

## Figures and Tables

**Table 1:** Dataset used for phylogenetic analysis. Genbank sequences include newly generated nrITS, nrLSU, and nrSSU amplicons relative to the novel species *Paralulworthia candida*, *P. elbensis*, and *P. mediterranea* (in bold).

Species	Strain	Source	nrITS	nrSSU	nrLSU
**Lulworthiales**					
**Lulworthiaceae**					
*Cumulospora marina* Schmidt	MF46	Submerged wood	–	GU252136	GU252135
	GC53	Submerged wood	–	GU256625	GU256626
*C. varia* Chatmala and Somrithipol	GR78	Submerged wood	–	EU848593	EU848578
	IT 152	Submerged wood	EU848579	EU848579	
*Halazoon mehlae* Abdel-Aziz, Abdel-Wahab and Nagah.	MF819 ^T^	Drift stems of *Phragmites australis*	–	GU252144	GU252143
*H. fuscus* (Schmidt) Abdel-Wahab, Pang, Nagah., Abdel-Aziz and Jones	NBRC 105256	Driftwood	–	GU252148	GU252147
*Hydea pigmaea* (Kohlm) Pang and Jones	NBRC 33069	Driftwood	–	GU252134	GU252133
	IT081	Driftwood	–	GU256632	GU256633
*Kohlmeyeriella crassa* (Nakagiri) Kohlm., Volkm.–Kohlm., Campb., Spatafora and Gräfenhan	NBRC 32133 ^T^	Sea foam	LC146741	AY879005	LC146742
*K. tubulata* (Kohlm.) Jones, Johnson and Moss	PP115	Marine environment	–	AY878998	AF491265
	PP0989	Marine environment	–	AY878997	AF491264
*Lindra marinera* Meyers	JK 5091	Marine environment	–	AY879000	AY878958
*L. obtusa* Nakagiri and Tubaki	NRBC 31317 ^T^	Sea foam	LC146744	AY879002	AY878960
	AFTOL 5012	Marine environment	–	FJ176847	FJ176902
	CBS 113030	n.d.		AY879001	AY878959
*L. thalassiae* Orpurt, Meyers, Boral and Simms	JK 5090A	Marine environment	–	U46874	U46891
	AFTOL 413	Marine environment	DQ491508	DQ470994	DQ470947
	JK 5090	Marine environment	–	AF195634	AF195635
	JK 4322	*Thalassia testudinum* leaves	–	AF195632	AF195633
*Lulwoana uniseptata* (Nakagiri) Kohlmeyer et al.	NBRC 32137 ^T^	Submerged wood	LC146746	LC146746	LC146746
	CBS 16760	Driftwood	–	AY879034	AY878991
*Zalerion maritima* (Linder) Anastasiou	FCUL280207CP1	Sea water	KT347216	KT347203	JN886806
	FCUL010407SP2	Sea water	KT347217	KT347204	JN886805
*Lulworthia atlantica* Azevedo, Caeiro and Barata	FCUL210208SP4	Sea water	KT347205	KT347193	JN886843
	FCUL190407CF4	Sea water	KT347207	KT347198	JN886816
	FCUL061107CP3	Sea water	KT347208	KT347196	JN886825
*L. fucicola* Sutherl.	ATCC 64288 ^T^	Intertidal wood	–	AY879007	AY878965
	PP1249	Marine environment	–	AY879008	AY878966
*L. grandispora* Meyers	AFTOL 424	Dead *Rhizophora* sp. branch	–	DQ522855	DQ522856
	NTOU3841	Driftwood	–	KY026044	KY026048
	NTOU3847	Decayed mangrove wood	–	KY026046	KY026049
	NTOU3849	Decayed mangrove wood	–	KY026047	KY026050
*Lulworthia lignoarenaria* (Koch and Jones) Kohlm., Volkm.–Kohlm., Campb., Spatafora and Gräfenhan	AFTOL 5013	Marine environment	–	FJ176848	FJ176903
*L. medusa* (Ellis and Everh.) Cribb and Cribb	JK 5581 ^T^	Spartina	–	AF195636	AF195637
*L. opaca* (Linder) Cribb and J.W. Cribb	CBS 218.60	Driftwood in seawater	–	AY879003	AY87896
*L.* cf. *purpurea* (Wilson) Johnson	FCUL170907CP5	Sea water	KT347219	KT347201	JN886824
	FCUL280207CF9	Sea water	KT347218	KT347202	JN886808
*Matsusporium tropicale* (Kohlm.) Jones and Pang	NBRC 32499	Submerged wood	–	GU252142	GU252141
*Moleospora maritima* Abdel-Wahab, Abdel-Aziz and Nagah.	MF 836 ^T^	Drift stems of *Phragmites australis*	–	GU252138	GU252137
***Paralulworthia candida* sp. nov.**	MUT 5430	*P. oceanica*	**MZ357724**	**MZ357767**	**MZ357746**
***Paralulworthia elbensis* sp. nov.**	MUT 377	*P. oceanica*	**MZ357710**	**MZ357753**	**MZ357732**
	MUT 5422	*P. oceanica*	**MZ357723**	**MZ357766**	**MZ357745**
	MUT 5438	*P. oceanica*	**MZ357712**	**MZ357755**	**MZ357734**
	MUT 5461	*P. oceanica*	**MZ357725**	**MZ357768**	**MZ357747**
*Paralulworthia gigaspora* Prigione, Poli, Bovio and Varese	MUT 435 ^T^	*P. oceanica*	MN649242	MN649246	MN649250
	MUT 5413	*P. oceanica*	MN649243	MN649247	MN649251
	MUT 263	Oil-contaminated sea water	**MZ357729**	**MZ357772**	**MZ357751**
	MUT 465	*P. oceanica*	**MZ357726**	**MZ357769**	**MZ357748**
	MUT 1753	Oil-contaminated sea water	**MZ357730**	**MZ357773**	**MZ357752**
	MUT 5085	*P. oceanica*	**MZ357715**	**MZ357758**	**MZ357737**
	MUT 5086	*P. oceanica*	**MZ357716**	**MZ357759**	**MZ357738**
	MUT 5093	*P. oceanica*	**MZ357718**	**MZ357761**	**MZ357740**
	MUT 5094	*P. oceanica*	**MZ357719**	**MZ357762**	**MZ357741**
*Paralulworthia halima* (Anastasiou) Gonçalves, Abreu and Alves	CMG 68	Submerged wood	MT235736	MT235712	MT235753
	CMG 69	Submerged wood	MT235737	MT235713	MT235754
	MUT 1483	Submerged wood	**MZ357727**	**MZ357770**	**MZ357749**
	MUT 2919	Submerged wood	**MZ357713**	**MZ357756**	**MZ357735**
	MUT 3347	Submerged wood	**MZ357728**	**MZ357771**	**MZ357750**
*Paralulworthia posidoniae* Poli, Prigione, Bovio and Varese	MUT 5261 ^T^	*P. oceanica*	MN649245	MN649249	MN649253
	MUT 5092	*P. oceanica*	**MZ357717**	**MZ357760**	**MZ357739**
	MUT 5110	*P. oceanica*	**MZ357720**	**MZ357763**	**MZ357742**
	MUT 5419	*P. oceanica*	**MZ357722**	**MZ35776**	**MZ357744**
***Paralulworthia mediterranea* sp. nov.**	MUT 654	*P. oceanica*	**MZ357711**	**MZ357754**	**MZ357733**
	MUT 5080	*P. oceanica*	**MZ357714**	**MZ357757**	**MZ357736**
	MUT 5417 ^T^	*P. oceanica*	**MZ357721**	**MZ357764**	**MZ357743**
**Pisorisporiales**					
**Pisorisporiaceae**					
*Achroceratosphaeria potamia* Réblová, Fourn. and Hyde	JF 08139 ^T^	Submerged wood of *Platanus* sp.	–	GQ996541	GQ996538
**Pleosporales**					
**Melanommataceae**					
*Bimuria novae-zelandiae* Hawksw., Chea and Sheridan	CBS 107.79 ^T^	Soil	MH861181	FJ190605	MH872950
**Pleosporaceae**					
*Setosphaeria monoceras* Alcorn	CBS 154.26	n.d.	DQ337380	DQ238603	AY016368
**Dydimosphaeriaceae**					
*Letendraea helminthicola* (Berk. and Broome) Weese ex Petch	CBS 884.85	Yerba mate	MK404145	AY016345	AY016362

^T^ = Type Strain.

**Table 2:** Genetic markers, primers, and thermocycler conditions used in this study.

	Forward and Reverse Primers	Thermocycler Conditions	References
**ITS**	ITS1–ITS4	95 °C: 5 min (95 °C: 40 s, 55 °C: 50 s, 72 °C: 50 s) × 35 cycles; 72 °C: 8 min; 4 °C: ∞	[19]
**LSU**	LR0R–LR7	95 °C: 5 min (95 °C: 1 min, 50 °C: 1 min, 72 °C: 2 min) × 35 cycles; 72 °C: 10 min; 4 °C: ∞	[20]
**SSU**	NS1–NS4	95 °C: 5 min (95 °C: 1 min, 50 °C: 1 min, 72 °C: 2 min) × 35 cycles; 72 °C: 10 min; 4 °C: ∞	[19]
**TEF-1α**	EF-dF/EF-2218R	95 °C: 5 min (95 °C: 1 min, 50 °C: 1 min; 72 °C: 2 min) × 40 cycles, 72 °C: 10 min; 4 °C: ∞	[21]
**βTUB**	Bt2a–Bt2b	94 °C: 4 min (94 °C: 35 s, 58 °C: 35 s, 72 °C: 50 s) × 35 cycles; 72 °C: 5 min; 4 °C: ∞	[22]
**RPB1**	RPB1Af–RPB1Cr	96 °C: 5 min (94 °C: 30 s, 52 °C: 30 s, 72 °C: 1 min) × 40 cycles; 72 °C: 8 min; 4 °C: ∞	[23]
**RPB2**	fRPB2-5F/fPB2-7cR	94 °C: 3 min (94 °C: 30 s; 55 °C: 30 s; 72 °C: 1 min) × 40 cycles, 72 °C: 10 min; 4 °C: ∞	[24]

## Data Availability

Sequence data are available at Genbank (NCBI) under the accession numbers reported in the manuscript.

## Supplementary Materials

The following are available online at https://www.mdpi.com/article/10.3390/jof7110940/s1: Figure S1, Phylogenetic inference based on a combined nrITS, nrSSU, nrLSU, RPB1, RPB2, TEF-1α, and βTUB dataset; Table S1, Dataset based on nrITS, nrLSU, nrSSU, RPB1, RPB2, TEF-1α, and βTUB used for phylogenetic analysis.
