# Supplementary material for: Insights on Lulworthiales Inhabiting the Mediterranean Sea and Description of Three Novel Species of the Genus Paralulworthia
Source: J Fungi (Basel). 2021 Nov 6;7(11):940. doi: 10.3390/jof7110940 (PMC8623521; doi:10.3390/jof7110940)
Supplement: Supplementary file 1 [file jof-07-00940-s001.zip › jof-1393551-supplementary.pdf]

Table S1. Dataset used for phylogenetic analysis. Genbank sequences include newly generated nrITS, nrLSU, nrSSU, RPB1, RPB2, TEF-1α and βTUB amplicons relative to the novel species *Paralulworthia candida*, *P. elbensis* and *P. mediterranea* (in bold)

Species	Strain	Source	nrITS	nrSSU	nrLSU	EF-1α	RPB1	RPB2	βTUB
**Lulworthiales**									
**Lulworthiaceae**									
Halazoon mehlae Abdel-Aziz, Abdel-Wahab & Nagah.	MF819 T	Drift stems of *Phragmites australis*	--	GU252144	GU252143	--	--	--	--
H. fuscus (Schmidt) Abdel-Wahab, Pang, Nagah., Abdel-Aziz & Jones	NBRC 105256	Driftwood	--	GU252148	GU252147	--	--	--	--
L. medusa (Ellis & Everh.) Cribb & Cribb	JK 5581 T	Spartina	--	AF195636	AF195637	--	--	--	--
L. cf. purpurea (Wilson) Johnson	FCUL170907CP5	Sea water	KT347219	KT347201	JN886824	--	--	--	--
	FCUL280207CF9	Sea water	KT347218	KT347202	JN886808	--	--	--	--
***Paralulworthia candida* sp. nov.**	MUT 5430	*P. oceanica*	**MZ357724**	**MZ357767**	**MZ357746**	**MZ407526**	**--**	**MZ357229**	**MZ407550**
***Paralulworthia elbensis* sp. nov.**	MUT 377	*P. oceanica*	**MZ357710**	**MZ357753**	**MZ357732**	**MZ407516**	**MZ365271**	**MZ357215**	**MZ407536**
	MUT 5422	*P. oceanica*	**MZ357723**	**MZ357766**	**MZ357745**	**MZ407525**	**--**	**MZ357228**	**MZ407549**
	MUT 5438	*P. oceanica*	**MZ357712**	**MZ357755**	**MZ357734**	**MZ407518**	**--**	**MZ357217**	**MZ407538**
	MUT 5461	*P. oceanica*	**MZ357725**	**MZ357768**	**MZ357747**	**--**	**--**	**MZ357230**	**--**
Paralulworthia gigaspora Prigione, Poli, Bovio & Varese	MUT 435 T	*P. oceanica*	MN649242	MN649246	MN649250	**MZ407533**	**MZ365286**	**MZ357236**	**MZ407557**
	MUT 5413	*P. oceanica*	MN649243	MN649247	MN649251	**MZ407534**	**MZ365287**	**MZ357237**	**MZ407558**
	MUT 263	Oil-contaminted Sea water	**MZ357729**	**MZ357772**	**MZ357751**	**MZ407531**	**--**	**MZ357234**	**MZ407555**
	MUT 465	*P. oceanica*	**MZ357726**	**MZ357769**	**MZ357748**	**MZ407534**	**MZ365282**	**MZ357231**	**MZ407552**
	MUT 1753	Oil-contaminted Sea water	**MZ357730**	**MZ357773**	**MZ357752**	**MZ407532**	**MZ365285**	**MZ357235**	**MZ407556**
	MUT 5085	*P. oceanica*	**MZ357715**	**MZ357758**	**MZ357737**	**MZ407521**	**MZ365274**	**MZ357220**	**MZ407541**
	MUT 5086	*P. oceanica*	**MZ357716**	**MZ357759**	**MZ357738**	**MZ407522**	**MZ365275**	**MZ357221**	**MZ407542**

	MUT 5093	*P. oceanica*	**MZ357718**	**MZ357761**	**MZ357740**	**--**	**MZ365277**	**MZ357223**	**MZ407544**
	MUT 5094	*P. oceanica*	**MZ357719**	**MZ357762**	**MZ357741**	**MZ407524**	**MZ365278**	**MZ357224**	**MZ407545**
Paralulworthia halima (Anastasiou) Gonçalves, Abreu & Alves	CMG 68	Submerged wood	MT235736	MT235712	MT235753	--	--	--	--
	CMG 69	Submerged wood	MT235737	MT235713	MT235754	--	--	--	--
	MUT 1483	Submerged wood	**MZ357727**	**MZ357770**	**MZ357749**	**MZ407529**	**MZ365283**	**MZ357232**	**MZ407553**
	MUT 2919	Submerged wood	**MZ357713**	**MZ357756**	**MZ357735**	**MZ407519**	**MZ365272**	**MZ357218**	**MZ407539**
	MUT 3347	Submerged wood	**MZ357728**	**MZ357771**	**MZ357750**	**MZ407530**	**MZ365284**	**MZ357233**	**MZ407554**
Paralulworthia posidoniae Poli, Prigione, Bovio & Varese	MUT 5261 ^{T}	*P. oceanica*	MN649245	MN649249	MN649253	**MZ407535**	**--**	**MZ357238**	**MZ407559**
	MUT 5092	*P. oceanica*	**MZ357717**	**MZ357760**	**MZ357739**	**MZ407523**	**MZ365276**	**MZ357222**	**MZ407543**
	MUT 5110	*P. oceanica*	**MZ357720**	**MZ357763**	**MZ357742**	**--**	**MZ365279**	**MZ357225**	**MZ407546**
	MUT 5419	*P. oceanica*	**MZ357722**	**MZ35776**	**MZ357744**	**--**	**MZ365281**	**MZ357227**	**MZ407548**
***Paralulworthia mediterranea* sp. nov.**	MUT 654	*P. oceanica*	**MZ357711**	**MZ357754**	**MZ357733**	**MZ407517**	**--**	**MZ357216**	**MZ407537**
	MUT 5080	*P. oceanica*	**MZ357714**	**MZ357757**	**MZ357736**	**MZ407520**	**MZ365273**	**MZ357219**	**MZ407540**
	MUT 5417 ^{T}	*P. oceanica*	**MZ357721**	**MZ357764**	**MZ357743**	**--**	**MZ365280**	**MZ357226**	**MZ407547**

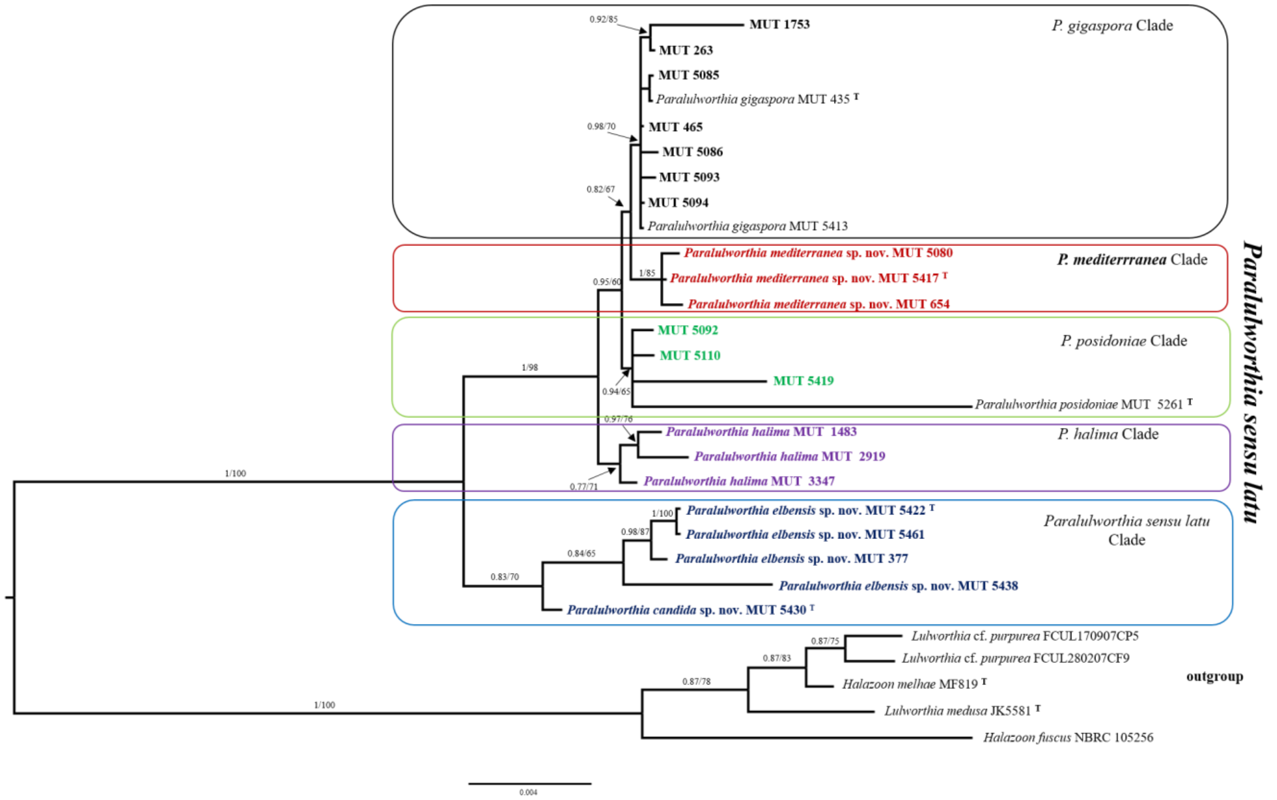

Figure S1. Phylogenetic inference based on a combined nrITS, nrSSU, nrLSU, RPB1, RPB2, TEF-1α and βTUB dataset. Colours indicate the belonging to different groups. Branch numbers indicate BYPP and BS values; T = Type Strain; Bar = expected changes per site (0.004).
